# Histone variants: dynamic punctuation in transcription

**DOI:** 10.1101/gad.238873.114

**Published:** 2014-04-01

**Authors:** Christopher M. Weber, Steven Henikoff

**Affiliations:** 1 Division of Basic Sciences, Fred Hutchinson Cancer Research Center, Seattle, Washington 98109, USA;; 2 Molecular and Cellular Biology Program, University of Washington, Seattle, Washington 98195, USA;; 3 Howard Hughes Medical Institute, Fred Hutchinson Cancer Research Center, Seattle, Washington 98109, USA

**Keywords:** gene regulation, histone chaperones, nucleosome dynamics, nucleosome remodeling

## Abstract

Eukaryotic gene regulation involves a balance between packaging of the genome into nucleosomes and enabling access to regulatory proteins and RNA polymerase. Nucleosomes are integral components of gene regulation that restrict access to both regulatory sequences and the underlying template. Whereas canonical histones package the newly replicated genome, they can be replaced with histone variants that alter nucleosome structure, stability, dynamics, and, ultimately, DNA accessibility. Here we consider how histone variants and their interacting partners are involved in transcriptional regulation through the creation of unique chromatin states.

## Abstract (precis)

Eukaryotic gene regulation involves a balance between packaging of the genome into nucleosomes and enabling access to regulatory proteins and RNA polymerase. Nucleosomes, consisting of DNA wrapped around a core of histone proteins, are integral components of gene regulation that restrict access to both regulatory sequences and the underlying template. In this review, Weber and Henikoff consider how histone variants and their interacting partners are involved in transcriptional regulation through the creation of unique chromatin states.

The basic unit of chromatin is the nucleosome, consisting of DNA wrapped around a core of histone proteins. Nucleosomes likely evolved to protect and compact increasingly large eukaryotic genomes (Malik and Henikoff 2003). As a consequence, however, nucleosomes also restrict access to cellular components such as DNA-binding transcription factors and RNA polymerase (Li et al. 2007). Accordingly, distinct mechanisms have evolved to influence the dynamic competition between nucleosomes and DNA-binding transcription factors in addition to orchestrating RNA polymerase II (RNAPII) translocation across a nucleosomal template. This dynamic mode of regulation is mediated in a number of ways, including post-translational modification of histones, altering the position or eviction of nucleosomes by ATP-dependent chromatin remodelers, and replacement of canonical histones with histone variants. Canonical histones (H2A, H2B, H3, and H4) are deposited in a replication-coupled manner to package the newly replicated genome. In contrast, histone variants are expressed throughout the cell cycle and replace canonical histones or take their place when nucleosomes are evicted (for reviews on evolutionary conservation, see Malik and Henikoff 2003; Talbert and Henikoff 2010). Histone variants have distinct amino acid sequences that can influence both the physical properties of the nucleosome and nucleosome dynamics. These properties are especially important during transcription, where histone variants shape the chromatin landscape of *cis*-regulatory and coding regions in support of specific transcription programs.

Here we consider core variants of H2A and H3 that are implicated in transcription (Table 1). We discuss the mechanisms responsible for shaping the histone variant landscape, focusing on recent genome-wide mapping studies of these variants, their chaperones, RNAPII, and nucleosome dynamics. We review how these chromatin landscapes and deposition pathways influence the dynamic interplay between nucleosome occupancy, regulatory DNA-binding proteins, and, ultimately, RNAPII elongation across nucleosomes. Our ultimate focus is on how histone variants create distinct chromatin landscapes with different dynamics and how this influences gene regulation.

**Table 1.**
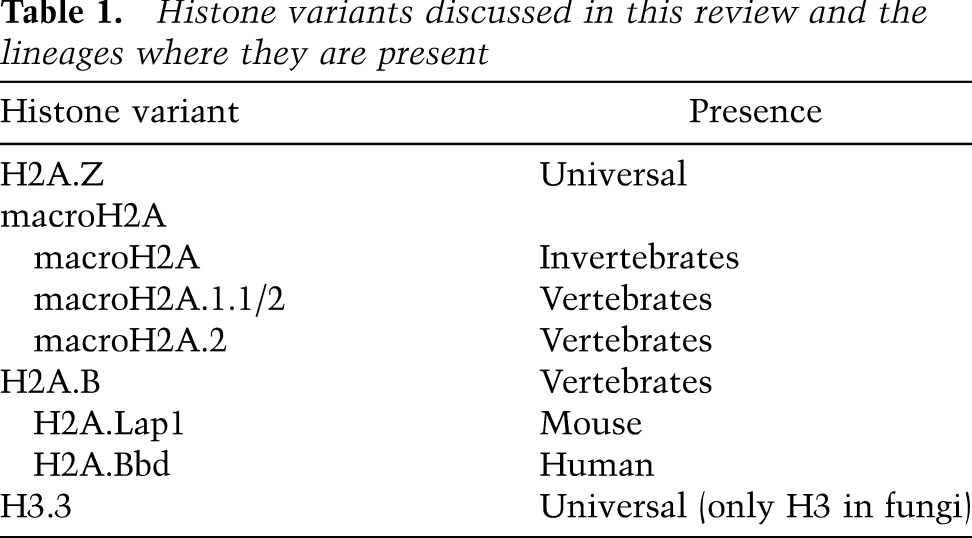
Histone variants discussed in this review and the lineages where they are present

## Nucleosome organization and dynamics

**Figure 1.**
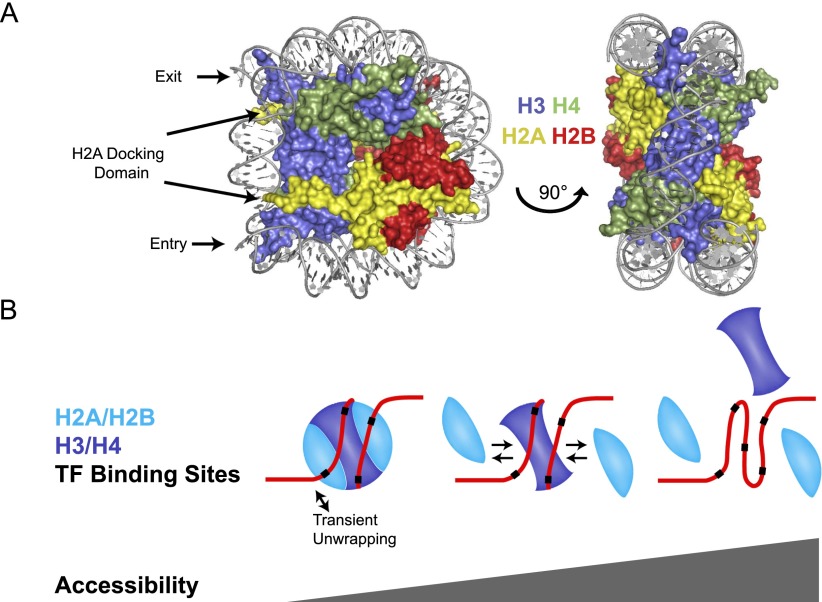
(*A*) Structure of nucleosome core particle, showing that (H3/H4)_2_ is at the center of the DNA wrap, with two dimers of H2A/H2B docked at the edges, near the DNA entry and exit locations. (*B*) Nucleosomes modulate access to transcription factor (TF)-binding sites. Transient DNA unwrapping exposes transcription factor-binding sites. As nucleosomes unwrap, H2A/H2B dimers can be lost, exposing more DNA, and when the nucleosome is completely unwrapped, (H3/H4)_2_ is lost, and DNA is completely exposed.

Each nucleosome wraps ∼147 base pairs (bp) of DNA 1.7 turns around an octamer consisting of two each of H2A, H2B, H3, and H4. At the center of the DNA wrap, an (H3/H4)_2_ tetramer is formed due to a strong four-helix bundle interaction between the two H3 proteins (Luger et al. 1997). Interacting with the (H3/H4)_2_ tetramer are two heterodimers of H2A/H2B, which dock at the DNA entry and exit sites through the H2A C terminus-docking domain (Fig. 1A). Additionally, the two H2A histones interact through their L1 loop, and H2B interacts with H4 through a weak four-helix bundle.

Nucleosomes are energetically stable; however, they can turn over in vivo (Fig. 1B). H2A and H2B turn over much faster than H3 and H4 in both genic and intergenic regions (Kimura and Cook 2001; Jamai et al. 2007). H2A/H2B turnover occurs due to weaker intranucleosomal contacts and because the dimers dock at DNA entry and exit sites, which are prone to transiently unwrap (Li et al. 2005b). Active processes such as RNA polymerase transit drive unwrapping, which increases dimer turnover and DNA exposure (Sheinin et al. 2013). A functional consequence of both transient unwrapping and dimer loss is exposure of DNA to binding by regulatory proteins.

In order for the (H3/H4)_2_ tetramer to turn over, the nucleosome must be almost completely unwrapped, a process that can occur many times at a given location during interphase. In yeast and *Drosophila* cells, nucleosome turnover measured by new H3 incorporation correlates with transcription (Dion et al. 2007; Deal et al. 2010). In *Drosophila* cells, nucleosome turnover is also high over *cis*-regulatory regions, consistent with a competition between nucleosomes and transcription factors for occupancy of these sites (Mito et al. 2007; Deal et al. 2010). A consequence of the dynamic nature of nucleosomes is erasure of post-translational modifications on histones. Thus, modulation of DNA accessibility through regulated nucleosome turnover can perpetuate gene expression states.

## Replication-independent deposition of variants punctuates the chromatin landscape

### H2A.Z alters nucleosome properties

H2A variants are the most diverse, perhaps reflective of relaxed structural constraint within the nucleosome. One such variant, H2A.Z, arose once early in eukaryotic evolution and has remained distinct from H2A ever since (Talbert and Henikoff 2010). At the amino acid level, H2A.Z is only ∼60% identical to H2A within species but is relatively conserved between species and is essential in metazoans (Zlatanova and Thakar 2008). Remarkably, the structure of the H2A.Z nucleosome is quite similar to H2A; however, there are key structural differences (Suto et al. 2000). On the surface, H2A.Z has an extended acidic patch, which stimulates remodeling activity with the ISWI ATP-dependent remodeler (Goldman et al. 2010). Within the core, the L1 loop is structurally distinct, and in the docking domain with H3/H4, a glutamine-to-glycine substitution in H2A.Z compromises three hydrogen bonds, which is predicted to weaken the interaction. Despite these structural differences, the change in the stability of the particle is subtle, with contrasting results reported. Overall, the consensus is that H2A.Z slightly stabilizes in vitro and destabilizes in vivo (Zlatanova and Thakar 2008; Bonisch and Hake 2012). This discrepancy might be attributable to post-translational modifications in vivo, where H2A.Z is acetylated at active genes (Bruce et al. 2005; Valdes-Mora et al. 2012); differences in DNA sequence; or the fact that H2A.Z nucleosomes can be hybrid in vivo, either heterotypic (Z/A) or homotypic (Z/Z) (Viens et al. 2006; Luk et al. 2010; Weber et al. 2010).

### H2A.Z deposition and a futile cycle

In yeast, H2A.Z can be bound by the general H2A/H2B chaperone Nap1 or Chz1, which preferentially bind H2A.Z over H2A (Luk et al. 2007). These chaperones provide a source of H2A.Z for the Swr1 remodeling complex, which exchanges H2A.Z for H2A (Mizuguchi et al. 2004). Some metazoans contain two Swr1 orthologs that organize into at least two distinct complexes, P400/TIP60 and SRCAP, which, like Swr1, catalyze the exchange reaction. While these complexes share some components, there are many differences (Billon and Cote 2012). For example, a human H2A.Z-specific chaperone, ANP32E, was recently characterized as part of P400/TIP60 but not the SRCAP complex (Obri et al. 2014). Intriguingly, the ANP32 family has many members of uncharacterized function, where at least one other, ANP32B, has been shown to be an H3/H4 chaperone (Tochio et al. 2010). It is likely that many other histone chaperones remain to be discovered.

**Figure 2.**
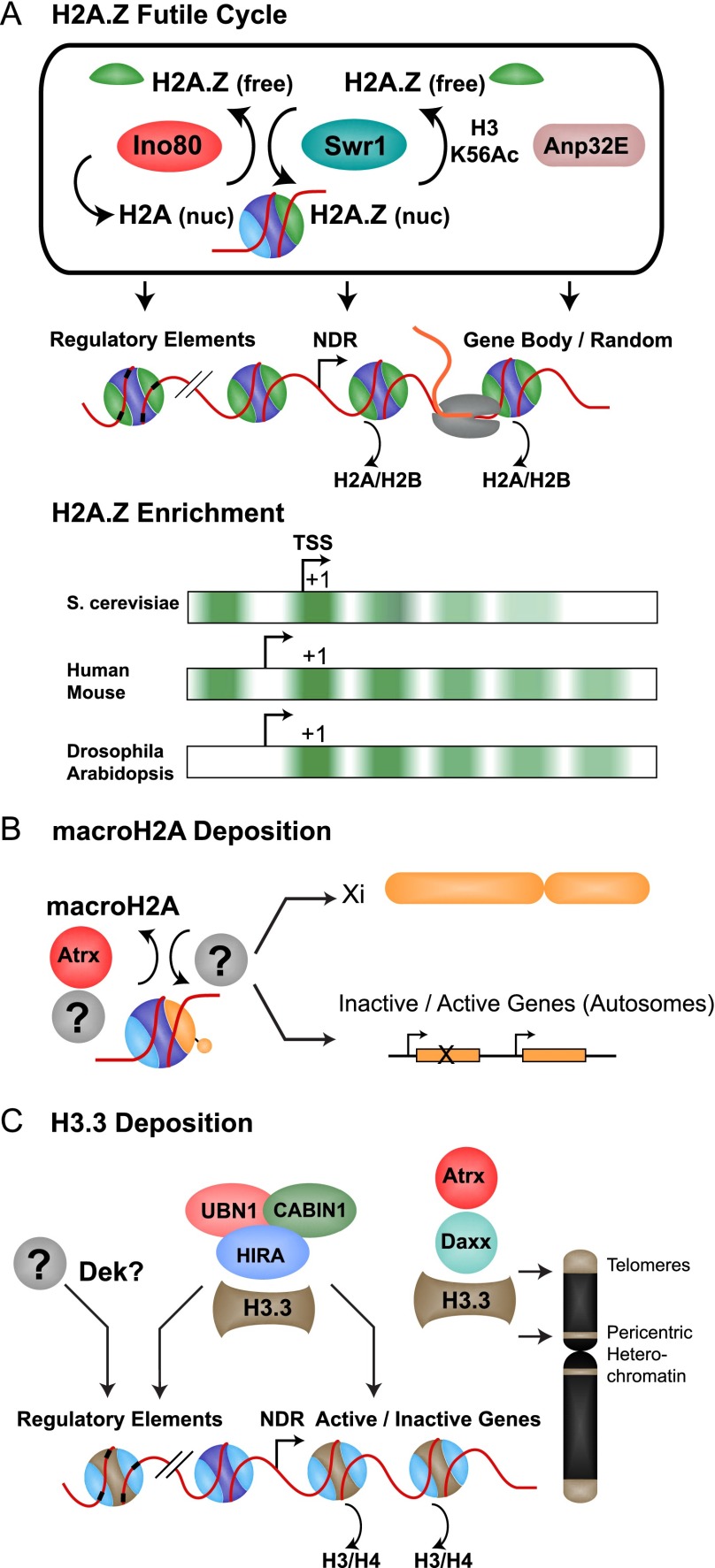
(*A*, *top*) The H2A.Z futile cycle of deposition by Swr1 or orthologous complexes and then removal by Ino80, Anp32E, or Swr1 when H3K56 is acetylated. (*Bottom*) H2A.Z enrichment patterns and promoter architecture differences in different organisms. (*B*) macroH2A is deposited in the inactive X (Xi) and inactive/active genes on autosomes. (*C*) H3.3 deposition is mediated by the HIRA complex at genes and regulatory elements, whereas Atrx and Daxx mediate H3.3 incorporation at telomeres and pericentric heterochromatin. It is not clear how H3.3 is enriched at some *cis*-regulatory elements.

H2A.Z comprises ∼15% of total H2A and is distributed throughout the genome nonrandomly in both euchromatin and heterochromatin, where it is monoubiquitinated (Sarcinella et al. 2007); however, it has remained unclear how H2A.Z becomes enriched at particular sites in the genome. High-resolution ChIP (chromatin immunoprecipitation) and biochemistry of the yeast Swr1 complex revealed that Swr1 preferentially acts at nucleosome-depleted regions (NDRs) that are >50–70 bp and requires the Swc2 subunit to bind DNA (Ranjan et al. 2013; Yen et al. 2013). NDRs are predominately located at the promoter region of active genes and are characterized by two well-positioned flanking nucleosomes. Certain transcription factors and chromatin remodelers are required for NDR establishment and maintenance, but H2A.Z and Swr1 are dispensable (Whitehouse et al. 2007; Hartley and Madhani 2009). Whereas recruitment to the NDR might be sufficient to explain H2A.Z enrichment at nucleosomes that flank promoters, H2A.Z is also enriched to some extent in gene bodies of all eukaryotes studied. Additionally, NDR recruitment does not explain how some organisms, including *Arabidopsis* (Zilberman et al. 2008) and *Drosophila* (Mavrich et al. 2008), lack upstream H2A.Z nucleosomes (Fig. 2A).

In metazoans, H2A.Z enrichment correlates with expression level. One attractive possibility is that dimer loss followed by replacement with H2A.Z contributes to genic enrichment patterns and low-level incorporation genome-wide. In support of this possibility, homotypic Z/Z nucleosomes are enriched over active genes (Weber et al. 2010; Nekrasov et al. 2012), which could be the result of transcription-mediated dimer loss and then opportunistic replacement with H2A.Z, which is produced throughout the cell cycle. Consistent with transcription-coupled replacement, the presence of upstream H2A.Z nucleosomes seen in some organisms correlates with bidirectional transcription in yeast and mammals (Core et al. 2008; Xu et al. 2009), whereas upstream H2A.Z enrichment is not seen in *Arabidopsis* and *Drosophila*. DNA methylation and H2A.Z are anti-correlated in plants and animals (Conerly et al. 2010; Zemach et al. 2010), and in *Arabidopsis*, there is mutual antagonism (Zilberman et al. 2008), suggesting that epigenetic factors also contribute to H2A.Z localization.

In mammalian cells, preferential H2A.Z localization also is seen at a subset of gene promoters associated with transcription factor binding (Gevry et al. 2007; Gallant-Behm et al. 2012). Additionally, work in embryonic stem cells (ESCs) has shown that H2A.Z preferentially localizes to promoters of silent genes occupied by the Polycomb-repressive complex 2 (PRC2) (Creyghton et al. 2008; Illingworth et al. 2012). H2A.Z is also enriched at enhancers in ESCs and facilitates the binding of PRC2 and H3K4me3 and H3K27me3 modifications (Hu et al. 2013). Also in ESCs, H2A.Z increases nucleosome accessibility at FoxA2-binding sites (Li et al. 2012) and transcription factor accessibility (Hu et al. 2013). Despite these intriguing observations, it remains unclear how H2A.Z becomes enriched at developmentally regulated regions.

H2AZ can be actively removed from chromatin. For example, the recently described ANP32E chaperone specifically removes H2A.Z from nucleosomes, including those at *cis*-regulatory sites (Obri et al. 2014). The Ino80 complex catalyzes replacement of H2A.Z with canonical H2A (Papamichos-Chronakis et al. 2011), just the reverse of the Swr1 complex, thus completing a futile cycle when both complexes act successively on the same nucleosome. In an Ino80 mutant, H2A.Z is globally mislocalized, suggesting that Ino80 clears out H2A.Z that is spuriously incorporated. Ino80 is also found at yeast NDRs, where high-resolution ChIP suggests that Nhp10, les5, and Arp8 subunits are important for binding, similar to the Swc2 subunit of the Swr1 complex (Yen et al. 2013). In addition, Swr1 can carry out the reverse reaction when H3K56 is acetylated in yeast (Watanabe et al. 2013). However, when Swc2 is present, this activity is inhibited, suggesting that this subunit functions as a lock to prevent further remodeling. In yeast, H3K56 is acetylated during replication-coupled nucleosome assembly and is present on ∼30% of total histone H3 (Xu et al. 2005), whereas it marks <1% in human cells (Xie et al. 2009). Considering its paucity in metazoans, it remains unclear how general a role H3K56ac plays in removal of H2A.Z.

### Other H2A variants: life on the edge

Unlike H2A.Z, which is nearly universal across eukaryotes, other replication-independent H2A variants implicated in transcription have evolved only in animals. For example, H2A.B, first described as H2A.Bbd (Barr body-deficient) has been found only in mammals. H2A.B is only ∼50% identical to H2A and is rapidly evolving (Ishibashi et al. 2010). The C terminus of H2A.B is 19 amino acids shorter than that of H2A, reducing the tail and part of the docking domain. H2A.B lacks an acidic patch on its surface and is also referred to as H2A.Lap1 (lacks acidic patch 1) (Soboleva et al. 2012). These modifications to the structure substantially alter the physical properties of H2A.B-containing nucleosomes. For example, human H2A.B nucleosome arrays inhibit the formation of compact chromatin fibers (Zhou et al. 2007). Mouse H2A.B can form partially compacted arrays due to a single aspartate residue in the acidic patch, which is not found in humans (Soboleva et al. 2012). Consistent with an integral role for the docking domain in modulating nucleosome stability, H2A.B nucleosomes are less stable, protect ∼30 bp less DNA against micrococcal nuclease digestion, and exchange much faster by FRAP than canonical H2A (Bao et al. 2004; Gautier et al. 2004; Doyen et al. 2006b; Bonisch and Hake 2012; Tolstorukov et al. 2012). These effects are largely attributable to the docking domain because adding the H2A C-terminal tail, including the docking domain, onto H2A.B partially reverses the instability (Doyen et al. 2006b).

Currently, it remains unclear whether there are specific chaperones or mechanisms for H2A.B deposition. However, the general chaperone NAP-1 can efficiently assemble and disassemble H2A.B dimers in vitro (Okuwaki et al. 2005). In HeLa cells, ectopically expressed H2A.B localizes over gene bodies and correlates with expression level (Tolstorukov et al. 2012). Similarly, in ESCs, endogenous H2A.B is enriched over the body of actively transcribed genes (Chen et al. 2014). In contrast, in mouse testis where H2A.B is especially abundant, it is enriched over gene promoters and lowly over gene bodies (Soboleva et al. 2012). It remains unclear why H2A.B localization is different; nonetheless, H2A.B deposition results in a distinct chromatin landscape that is destabilized and less compact.

Another H2A variant implicated in transcription, macroH2A, is distinct from all other histones in that it contains a nonhistone globular (macro) domain. The macro domain on the C terminus is connected through an unstructured linker to a histone fold domain that is ∼60% identical to canonical H2A, resulting in a histone protein that is approximately three times the size of H2A (Chakravarthy et al. 2005). Macro domain-containing proteins are found in many organisms; however, macroH2A is restricted to vertebrates and a few invertebrates (Talbert and Henikoff 2010). Macro domains are known to bind metabolites of NAD^+^, including poly(ADP-ribose), and have distinct biological roles, including transcriptional regulation (Han et al. 2011). In vertebrates, there are three macroH2A isoforms: macroH2A.1.1, macroH2A.1.2, and macroH2A.2. The first two are splice isoforms from a single gene, whereas a separate gene encodes the latter. However, only macroH2A.1.1 is capable of binding metabolites of NAD^+^. In contrast to both H2A.Z and H2A.B, macroH2A preferentially forms heterotypic nucleosomes in vitro, whereas H2A.Z shows no preference (Chakravarthy et al. 2004; Chakravarthy and Luger 2006). macroH2A has a higher salt-dependent stability than H2A, where four residue changes in the L1 loop are most important (Abbott et al. 2005; Chakravarthy and Luger 2006).

In vivo, macroH2A is enriched on the transcriptionally inactivated female X chromosome, senescence-associated heterochromatic foci (SAHF), and large transcriptionally silent domains (Costanzi and Pehrson 1998; Zhang et al. 2005; Gamble et al. 2010; Tolstorukov et al. 2012). macroH2A localization to the inactive X is disrupted when Xist RNA is deleted, suggesting a role for this essential *cis*-regulatory RNA in macroH2A recruitment (Csankovszki et al. 1999). Both the histone and nonhistone segments of macroH2A are sufficient for targeting to the inactive X (Chadwick et al. 2001; Nusinow et al. 2007). In SAHF, macroH2A deposition is promoted by histone regulator A (HIRA), a chaperone responsible for histone variant H3.3 incorporation at genes, and the nucleosome assembly and disassembly factor Asf1 (Zhang et al. 2005). Rather than directly deposit macroH2A, these chaperones might help to clear the way for macroH2A deposition. Consistent with higher stability and a generally repressive role, macroH2A appears to impair transcription factor binding and has been suggested to impair remodeling by SWI/SNF and ISWI (Angelov et al. 2003). Although more recent results suggest that macroH2A is an excellent substrate for remodeling by these remodelers, macroH2A reduces recruitment of the SWI/SNF remodeler (Chang et al. 2008). macroH2A also associates with the SWI/SNF family DNA translocase ATRX (α-thalassemia/MR, X-linked), which, like Ino80 for H2A.Z, negatively regulates macroH2A deposition (Fig. 2B; Ratnakumar et al. 2012). Despite identification of these partners for macroH2A, it remains unclear how it is localized into specific genomic regions.

### H3.3: filling gaps and more

When a nucleosome is lost independent of replication, the (H3/H4)_2_ tetramer is replaced by the H3.3 variant and its H4 partner. Most eukaryotes express canonical H3 for replication-coupled deposition and the replication-independent H3.3 variant; however, some eukaryotes, such as fungi, express only the H3.3 type (Malik and Henikoff 2003). In metazoans, H3.3 differs from H3 by only four to five amino acids (Filipescu et al. 2013). Three of these differences are found within the core histone fold domain and specify the alternative deposition pathways (Ahmad and Henikoff 2002). The fraction of the genome occupied by H3.3 is variable, depending largely on dilution by canonical H3 during replication. For example, H3.3 comprises ∼90% of the histone 3 in terminally differentiated neurons (Pina and Suau 1987); however, in dividing cells, H3.3 comprises only ∼20% (McKittrick et al. 2004). H3.3 incorporation is largely opportunistic, occurring when DNA is exposed at dynamic regions such as gene promoters, the body of active genes, and *cis*-regulatory elements (Mito et al. 2007; Ray-Gallet et al. 2011; Schneiderman et al. 2012). H3.3 was also found to be enriched over a subset of repressed genes in mammalian ESCs that exhibit lower dynamics, suggesting an expanded role for H3.3 beyond simply filling gaps in active chromatin (Goldberg et al. 2010). H3.3 enrichment over genes, both active and inactive, depends on the HIRA complex (Tagami et al. 2004). In addition, HIRA-independent mechanisms of H3.3 incorporation have been described (Banaszynski et al. 2013). For example, Daxx has been identified as a novel H3.3-specific chaperone, which, together with the SWI/SNF family remodeler Atrx, is responsible for incorporation at telomeres and pericentric heterochromatin (Drane et al. 2010; Goldberg et al. 2010). A single methionine-to-glycine substitution at position 90 in H3.3 appears to be a dominant contributor to the specificity of H3.3 interaction with the Daxx chaperone (Elsasser et al. 2012). In *Drosophila*, Daxx and Dek have been shown to deposit H3.3 in regulatory elements (Sawatsubashi et al. 2010).

From a structural perspective, H3 occupies the center of the nucleosome, and so it might not be surprising that the sequence of an H3 variant would be more constrained than is seen for variants of H2A. However, this constraint is not observed in the centromere-specific H3 variant cenH3, which shares only ∼50%–60% identity with H3 within the histone fold domain (Malik and Henikoff 2003). Considering that the alterations to H3.3 are subtle, it is not surprising that no destabilization attributable to H3.3 nucleosomes has been detected in vitro (Thakar et al. 2009; Chen et al. 2013). However, analysis of chromatin from chicken cells found that H3.3-containing nucleosomes are more sensitive to salt-dependent disruption and that H3.3/H2A.Z double-variant nucleosomes were most unstable (Jin and Felsenfeld 2007). This effect was independent of acetylation, a modification associated with destabilization of nucleosomes, suggesting that the effect is intrinsic or is due to incorporation at active regions of the genome. In support of the latter explanation, H3.3/H2A.Z nucleosomes are enriched over regulatory elements and NDRs, which are frequently disrupted (Jin et al. 2009). However, nucleosome turnover and HIRA binding to chromatin are reduced after H3.3 depletion (Banaszynski et al. 2013). HIRA was recently shown to directly interact with transcription factors and the Brg1 chromatin remodeling complex (Pchelintsev et al. 2013). These results overall suggest that incorporation of H3.3 promotes a hyperdynamic state through its interaction partners within the nucleus.

## Histone variants in transcriptional regulation

### H2A.Z: the positive, the negative, and the unknown

**Figure 3.**
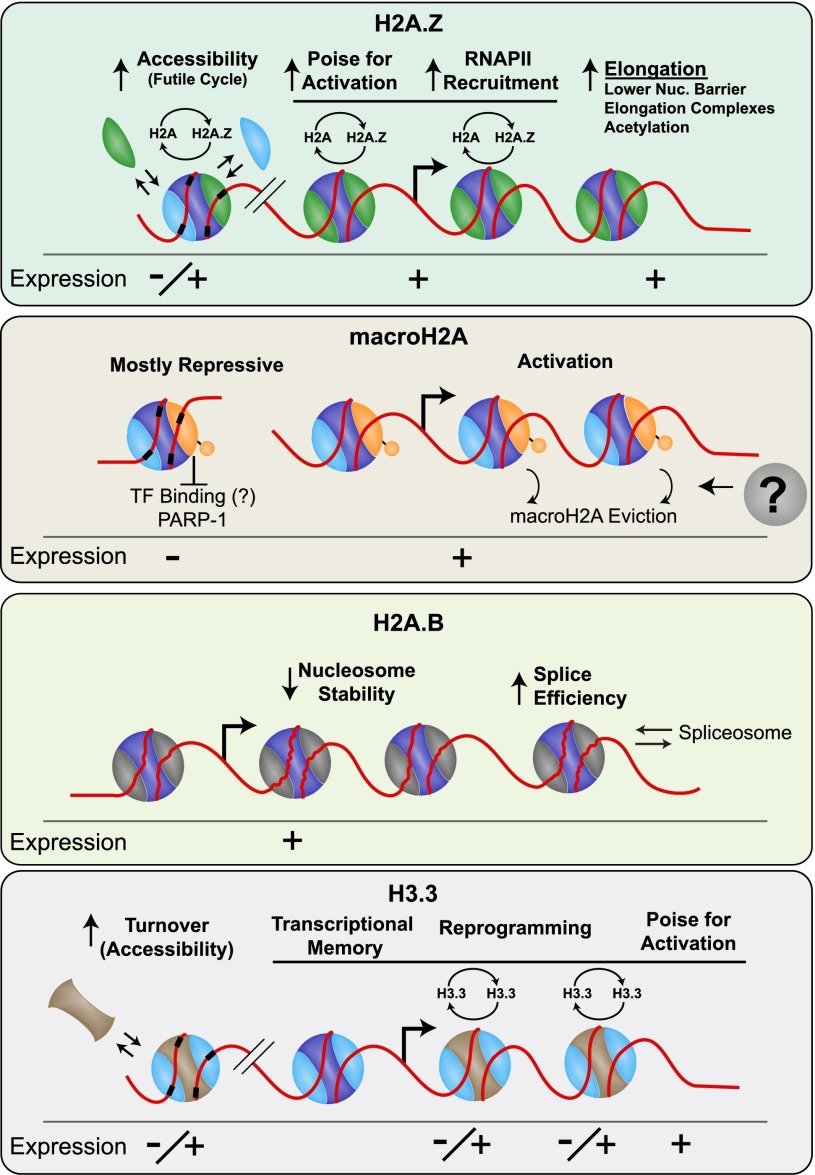
Models to explain the general role of histone variants and their deposition pathways on transcriptional regulation. Plus or minus expression level denotes the effect of the variant on transcriptional output.

A role for H2A.Z in transcription was initially proposed >30 years ago with the observation that *Tetrahymena* H2A.Z is present in the transcriptionally active macronucleus but not in the inactive micronucleus (Allis et al. 1980). Initial studies investigating the effect of H2A.Z on transcription were conducted in yeast, where it was shown to antagonize telomeric silencing, interact with activators, and help recruit RNAPII (Santisteban et al. 2000; Adam et al. 2001; Meneghini et al. 2003). Upon activation, H2A.Z is lost from 5′ ends of genes, suggesting that it poises genes for activation by enabling access to the promoter/transcription start site (TSS). In human cells, H2A.Z is exchanged prior to RNAPII loading, likely as a consequence of promoter remodeling, where it has a role in RNAPII recruitment (Hardy et al. 2009). It is possible that the difference in the action of H2A.Z between yeast and metazoans stems from the fact that, in yeast, there is a nucleosome over the TSS that must be removed for RNAPII to load (Fig. 2A; Rhee and Pugh 2012). Regardless of the nucleosome architecture at promoters, H2A.Z consistently plays an activating role (Fig. 3).

In addition to roles in activation and initiation, H2A.Z has been found to promote elongation (Santisteban et al. 2011). To explore the molecular basis for this role, we developed a single-base-resolution method to map the position of RNAPII through the 3′ end of nascent transcripts (3′ NT) in *Drosophila*. We found an anti-correlation between H2A.Z occupancy and RNAPII stalling as it transcribes across nucleosomes genome-wide (Weber et al. 2014). When H2A.Z levels were reduced both directly and by impairing Swr1 activity, the nucleosome barrier to RNAPII increased. Our results favor a model in which H2A.Z/H2B dimers are more easily lost when the nucleosome is unwrapped, aiding RNAPII transcriptional elongation. It is also possible that elongation factors such as the FACT complex have increased activity with H2A.Z nucleosomes to ease the barrier or that acetylation “unlocks” H2A.Z nucleosomes by destabilizing them. For example, in yeast, H2A.Z and Spt16, a component of the FACT complex, are synthetic-lethal (Biswas et al. 2006). Also, H2A.Z is hyperacetylated only when genes are expressed (Bruce et al. 2005; Valdes-Mora et al. 2012), and acetylation is required to achieve proper transcript levels (Halley et al. 2010; Valdes-Mora et al. 2012).

Despite a generally positive role for H2A.Z in transcription, negative effects have also been reported. For example, yeast h2a.zΔ mutants derepress silencing at the HMR locus (Dhillon and Kamakaka 2000), and whole-genome transcriptome analysis identified both positive and negative changes (Meneghini et al. 2003). In mammals, H2A.Z negatively regulates p21 and targets of the p63 transcription factor and occupies the promoters of genes that are silenced during mitosis (Gevry et al. 2007; Kelly et al. 2010; Gallant-Behm et al. 2012). Intriguingly, the h2a.zΔ phenotype, which extends beyond transcription defects, is partially suppressed by also mutating integral components of the Swr1 complex (Halley et al. 2010; Morillo-Huesca et al. 2010). This suggests that Swr1 activity in the absence of H2A.Z causes “mischief” by removing H2A but is unable to replace it. In the absence of both H2A.Z and Swr1, more yeast genes are down-regulated than up-regulated (Morillo-Huesca et al. 2010). However, in *Arabidopsis*, h2a.zΔ swr1Δ (pie1-5) mutants are worse off than either individual mutant, suggesting nonredundant functions for Swr1 (Coleman-Derr and Zilberman 2012). In *Arabidopsis* mutants that cannot incorporate H2A.Z, there is also global misregulation of transcription, with many genes up-regulated and many down-regulated. Interestingly, in *Arabidopsis*, H2A.Z is lost at elevated temperatures independent of transcription, and the transcriptome of the incorporation mutant resembles that of temperature-shifted plants (Kumar and Wigge 2010). Thus, in both yeast and plants, where H2A.Z mutants are viable, loss of the H2A.Z deposition pathway results in global effects on transcription.

How might H2A.Z function to exert both positive and negative effects on transcription? An attractive explanation is that H2A.Z facilitates binding of both activating and repressive complexes by keeping regions of the genome accessible. Support for this model comes from work in ESCs and during differentiation that has shown that H2A.Z facilitates binding of PRC2, MLL, and transcription factors (Creyghton et al. 2008; Li et al. 2012; Hu et al. 2013). In this context, H2A.Z deposition increases the fraction of the genome that is nuclease-hypersensitive and decreases nucleosome occupancy at enhancers (Hu et al. 2013). Interestingly, nucleosome depletion at transcription factor-binding sites is dependent on SWI/SNF and Ino80 chromatin remodeling complexes (Li et al. 2012). This suggests that a function of the futile cycle of deposition and removal might be to modulate accessibility to various regulatory proteins. It was previously suggested that H2A.Z regulates nucleosome positioning around promoters, which could influence accessibility of *cis*-regulatory regions (Guillemette et al. 2005; Marques et al. 2010). However, we and others failed to detect changes in nucleosome positioning following H2A.Z knockdown or deletion (Li et al. 2005a; Hartley and Madhani 2009; Weber et al. 2014). Although there might be intrinsic effects of H2A.Z on translational or rotational positioning in yeast (Albert et al. 2007), it is also possible that these attributes are due to enriching for promoter-flanking nucleosomes, having little to do with the properties of H2A.Z.

Overall, H2A.Z functions to support transcriptional activation and elongation, which helps to explain its enrichment near promoters and over the coding region of most genes in eukaryotes. Future work will determine which components are involved in H2A.Z dynamics and how these accessible regions are regulated both positively and negatively. This is especially relevant in mammalian cells, which deposit H2A.Z through P400 and SRCAP complexes, whose independent functions are not yet clear. That H2A.Z functions to facilitate access to both repressive and active regulatory complexes explains the vexing dual nature of H2A.Z in transcription and helps to explain why H2A.Z is essential in development.

### H2A.B

Whereas the role of H2A.B in transcription has not been as thoroughly investigated as that of H2A.Z, recent experiments have provided interesting results. H2A.B knockdown in HeLa cells resulted in substantial changes in gene expression, with more genes down-regulated than up-regulated (Tolstorukov et al. 2012). In mouse ESCs, H2A.B knockdown resulted in a more modest effect on gene expression; however, most genes were down-regulated. Included in the differentially expressed set are a few imprinted genes where it was shown that H2A.B facilitates elongation over the differentially methylated region of the gene (Chen et al. 2014). Consistent with a role for H2A.B in transcriptional elongation, H2A.B has also been shown to associate with components of the spliceosome, and, upon knockdown, RNA splicing is less efficient (Tolstorukov et al. 2012). In mouse testis, H2A.B is associated with promoter regions, as discussed above, although it is not yet known whether it has any specific effect on expression (Soboleva et al. 2012). Overall, H2A.B appears to promote transcriptional elongation, likely as a consequence of creating less-stable nucleosomes that are more easily disrupted upon interaction with RNAPII (Fig. 3).

### macroH2A

macroH2A localization on the inactivated female X chromosome, silent SAHF, and large transcriptionally silent domains (Costanzi and Pehrson 1998; Zhang et al. 2005; Gamble et al. 2010; Tolstorukov et al. 2012) suggests a role in transcriptional repression, although macroH2A is not required for X-chromosome inactivation (Changolkar et al. 2007). Genome-wide studies in human NT2 cells have shown that macroH2A is enriched at developmentally regulated genes, including overlap with the PRC2 complex. Nevertheless, it also represses transcription in vitro (Doyen et al. 2006a); in vivo, it represses IL8 transcription in a human B-cell line and endogenous retroviruses in mice (Agelopoulos and Thanos 2006; Changolkar et al. 2008). Knockdown of macroH2A increased the sensitivity of genes in the HOXA cluster to retinoic acid, further suggesting that it acts to repress transcription (Buschbeck et al. 2009). In the human breast cancer cell line MCF-7, most genes with macroH2A enrichment are not expressed; however, macroH2A depletion did not cause their up-regulation. Somewhat surprisingly, activating roles have also been reported for macroH2A, such as serum starvation-induced genes (Fig. 3; Gamble et al. 2010). Currently, the mechanism for this dual role of macroH2A remains unclear. It is possible that some of these discrepancies can be explained by macroH2A.1.1 inhibition of PARP-1 (Ouararhni et al. 2006) or poly(ADP-ribose) modification, both of which are known to be involved in transcriptional regulation.

### H3.3: a dynamic memory

The function of H3.3 in transcription remains somewhat unclear. Studies in *Tetrahymena* showed that H3.3 is not essential for transcription or viability (Cui et al. 2006). In adult *Drosophila* males, the loss of both H3.3 genes results in partial lethality and mostly affects highly expressed genes, with more genes up-regulated than down-regulated overall (Sakai et al. 2009). However, constitutive expression of H3 largely rescued these effects, suggesting that transcription differences were a consequence of nucleosome depletion and not specifically H3.3. In H3.3-depleted or HIRA^−/−^ ESCs, a minor fraction of genes showed differences in transcript levels, some of which are developmentally regulated; however, there is a much larger effect when H3.3 is depleted in partially differentiated mouse embryonic fibroblasts (Goldman et al. 2010; Banaszynski et al. 2013).

Recent evidence suggests that there are unique roles for H3.3 and its interacting partners in transcription. For example, H3.3 is important for early gene activation in cell lines and during myogenic differentiation (Placek et al. 2009; Tamura et al. 2009; Yang et al. 2011). In *Xenopus*, HIRA-mediated deposition of H3.3 is required for the transcriptional memory of active genes after somatic cell transfer into enucleated eggs (Ng and Gurdon 2008; Jullien et al. 2012). H3.3 has also been shown to prime genes for later activation after genotoxic stress (Adam et al. 2013). Although the molecular basis for transcriptional memory is unknown, it is intriguing that H3.3/H4 tetramers at human enhancer elements split during replication (Huang et al. 2013). H3.3 deposition might facilitate transcription factor binding by keeping these regions of the genome accessible (Fig. 3). Experiments in ESCs also support this general role, where H3.3 knockdown compromised PRC2 binding as well as H3K27me3 at “bivalent promoters” (Banaszynski et al. 2013). A similar role has been ascribed to H2A.Z, and it is thus conceivable that there is some cross-talk or that increased H2A.Z dynamics influence H3.3 deposition and dynamics.

## Perspective

Over the past few years, there has been a growing appreciation for histone variants in transcriptional regulation. From the evidence described in this review, chromatin-mediated gene regulation acts primarily through modulation of nucleosome dynamics and access to the underlying DNA. Although variants are structurally distinct from their canonical counterparts, replacement of a canonical histone with a variant is not the only way to alter the stability and dynamics of nucleosomes. An emerging theme is that variants function as an ensemble, coordinately modifying nucleosome properties and interacting with an expanding catalog of other factors within the nucleus. Differences in physical properties of variants and interactions with *trans*-acting factors result in the dynamic punctuation of chromatin that profoundly influences accessibility of the genome and, ultimately, transcriptional regulation. This is perhaps most evident during development in metazoans, which involves global changes in chromatin organization and transcriptional programs. A major area of interest for the future will be the characterization of metazoan deposition complexes, where we anticipate many context-dependent roles and the emergence of many additional players.
